# Prevalence, incidence, and trends of epilepsy among children and adolescents in Africa: a systematic review and meta-analysis

**DOI:** 10.1186/s12889-024-18236-z

**Published:** 2024-03-12

**Authors:** Gebeyaw Biset, Nigusie Abebaw, Natnael Atnafu Gebeyehu, Natan Estifanos, Endalk Birrie, Kirubel Dagnaw Tegegne

**Affiliations:** 1 Department of Pediatrics and Child Health Nursing, School of Nursing and Midwifery, College of Medicine and Health Sciences, Wollo University, Dessie, P.O.BOX: 1145, Ethiopia; 2 Department of Nursing, College of Medicine and Health Sciences, Wollo University, Dessie, Ethiopia; 3 Department of Midwifery, School of Nursing and Midwifery, College of Medicine and Health Sciences, Wollo University, Dessie, Ethiopia; 4 School of Midwifery, College of Health Sciences and Medicine, Wolaita Sodo University, Wolaita Sodo, Ethiopia

**Keywords:** Epilepsy, Children and adolescents, Prevalence, Africa

## Abstract

**Background:**

Epilepsy contributes to a significant disease burden in children and adolescents worldwide. The incidence of childhood epilepsy is threefold higher in low and middle income countries compared in high-income countries. Epilepsy is a serious neurological condition associated with stigma and discrimination, an impaired quality of life, and other mental health related problems.

**Objective:**

This study is aimed to synthesize existing evidence and estimate the pooled prevalence and incidence of epilepsy in children and adolescents in Africa.

**Methods:**

A comprehensive and systematic search of relevant databases was conducted. The quality of each study was assessed using the Newcastle-Ottawa Quality Assessment Scale adapted for meta-analysis. Two reviewers screened retrieved articles, conducted critical appraisals, and extracted the data. Heterogeneity between studies was assessed by visual inspection of forest plots and statistically using Cochran’s Q statistics and the I^2^ test. Publication bias was checked by visual inspection of funnel plots as well as statistically using Egger’s correlation and Begg’s regression tests. Finally, the pooled prevalence and incidence of childhood epilepsy were computed with 95% confidence intervals.

**Result:**

In this review and meta-analysis 42 studies with 56 findings were included to compute the pooled prevalence of childhood epilepsy. On the other hand, 6 studies were included to estimate the combined incidence. The pooled prevalence of cumulative epilepsy was 17.3 per 1000 children. Whereas the pooled prevalence of active and lifetime epilepsy was 6.8 and 18.6 per 1000 children respectively. The pooled incidence of childhood epilepsy was 2.5 per 1000 children.

**Conclusion:**

Nearly 1 in 50 children are suffering from epilepsy in Africa. However, little attention has been paid to the prevention and treatment of childhood epilepsy. Mass epilepsy screening, scaling up treatment coverage, and designing strict treatment follow up and monitoring mechanisms are recommended.

## Introduction

Epilepsy is a chronic neurological condition that affects over 50 million people of all ages and sexes worldwide [1–3]. The prevalence of epilepsy is disproportionately concentrated in low and middle-income countries (LMICs). The incidence of epilepsy is almost threefold higher in LMICs (139 per 100, 000) compared in high-income countries (HICs) (48.9 per 100, 000) [4–6]. Moreover, premature mortality associated with epilepsy is significantly higher in LMIC compared in HICs. The high burden of epilepsy in LMICs is largely attributed to inadequate medical services, poor socioeconomic conditions, and traditional beliefs regarding the treatment of epilepsy [7].

Epilepsy contributes to a significant disease burden in children and adolescents worldwide. Globally, more than 11 million children aged less than 15 years have active epilepsy [8–10]. In 2017, more than 291 million children aged less than 20 had epilepsy and intellectual disabilities, of which 95% lived in low and middle income countries [11]. In addition, more than 90% of epileptic cases in Sub-Saharan countries have been reported in children and adolescents aged < 20 years [12, 13]. Similarly, majority of epileptic cases in Ethiopia are reported in children and young adolescents [14, 15].

The highest incidence of epilepsy is reported during the early age of children and decline as age increases to adulthood [5, 16, 17]. A study in Norway suggests that incidence of epilepsy was reported144 per 100, 000 person-years in the first year of life. It then dropped to 61 per 100, 000 person-years in children aged 1 to 4 years and 54 per 100, 000 person-years in children aged 5 to 10 years. The perinatal and neonatal complications as well as early childhood infection contributes to the high burden of epilepsy in the early age of children [16].

Epilepsy is caused by both modifiable and non-modifiable risk factors. However, the causes are varied significantly between developing and developed countries. The main etiologies of epilepsy in developing countries are birth asphyxia, febrile seizures, perinatal and neonatal problems, and head related traumas. Whereas the common etiologies reported in developed countries are brain tumors, traumatic head injury, and cerebrovascular diseases [7, 12, 18]. In addition, epilepsy is found to be higher in children with family history of seizure. More than 20% of childhood epilepsy is associated with genetic inheritance [19]. Furthermore, parasitic infections like onchocerca volvulus, neurocysticercosis, and infection with plasmodium species are associated with increased rate of childhood epilepsy [20].

Epilepsy has a deadly impact on the lives of children and their family members. It is the leading cause of neurological impairment in children worldwide [21]. Children with epilepsy experiences poor school performance and school dropout, traumas including head traumas and burns, stigma and discriminations, mental health problems, impaired cognitive development, and premature mortality [22, 23]. In addition, epilepsy causes serious psychosocial consequences among families members due to the belief that the condition is resulted from sorcery practiced by the society, a breach of traditional cultural taboos, bad luck, or punishment from God for wrong feat [3, 24, 25].

Although epilepsy has such enormous negative consequences on the lives of children and their families; little or no attention has been paid to the treatment and prevention of disease in Africa. Consequently, millions of children and their families are still suffering from epilepsy. Additionally, no conclusive studies have been conducted to show the burden of epilepsy among children in Africa. Therefore, the findings of this study provide more general and conclusive evidence that provides essential insights to the prevention and treatment of childhood epilepsy in the continent.

## Method

### Design and protocol registration

A systematic review and meta-analysis of observational studies on childhood epilepsy were conducted in the African countries. The protocol for this review and meta-analysis was prepared in accordance with the Preferred Reporting Items for Systematic Review and Meta-analysis Protocols (PRISMA-P-2015) statement [26]. The protocol is registered at PROSPERO with the registration number CRD42022329754/www.crd.york.ac.uk/prospero.

### Eligibility criteria

The inclusion criteria for this study were studies on epilepsy among children aged less than 18 years; studies conducted in the African countries; observational studies (case-control, cross-sectional, and cohort studies); studies reported the prevalence or incidence of epilepsy; and English language articles that have been published in peer-reviewed journals without restriction of publication date. Articles that were not fully accessed were excluded because of incomplete data. Additionally, case reports, conference reports, expert opinions and qualitative studies were excluded.

### Search strategies

Systematic search of electronic databases MEDLINE/PubMed, HINARI, Web of Science, SCOPUS, and African Journals online (AJOL), as well as other gray literature and online open-access institutional repositories were retrieved using different search strategies. In addition, manual searches were conducted to identify additional studies. Manual searches are supplemental approaches to database searches conducted by inputting specific search terms or conditions into a search system or interface [27]. The searching terms were developed based on the research questions and study objectives. The following keywords were used in combination or in separation to find relevant articles in the African countries:

Epilepsy/Seizure/Convulsion, and Magnitude/Epidemiology/Prevalence/Incidence, and Children/Adolescents/Pediatrics/Infants/ Neonates.

### Study screening and selection

**Fig. 1:**
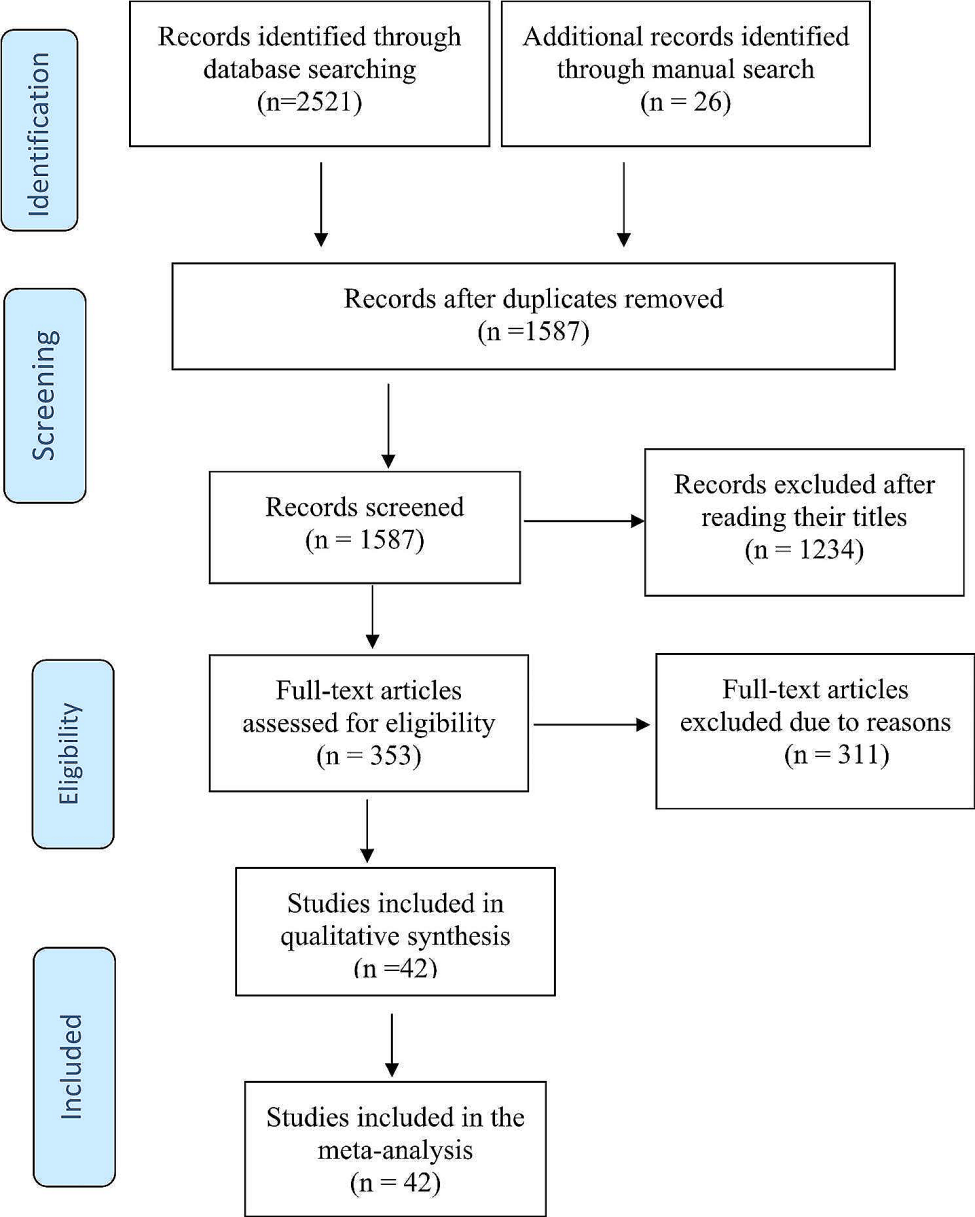
The Preferred Reporting Items for Systematic Reviews and Meta-Analyses (PRISMA) flow chart to screen studies included in the review and meta-analysis

The Preferred Reporting Items for Systematic review and Meta-analyses (PRISMA-2015) diagram was used to screen retrieved studies and report the findings [28]. A total of 2547 studies were retrieved through electronic databases, gray literature, and manual searches. All retrieved articles were imported to EndNote X8 reference managers and 960 articles were removed because of duplications. After excluding duplicate articles, the titles and abstract of 1587 articles were reviewed and 1234 were removed because of unrelated titles or outcomes not reported. The full text of 353 articles were reviewed which resulted in further exclusion of 311 articles. Finally, 42 articles with 56 epilepsy reports (unclassified, active, or lifetime epilepsy) were included (Fig. 1).

### Quality assessment

The quality of each study was assessed using the Newcastle-Ottawa Quality Assessment Scale (NOS) adapted for meta-analysis. Stars were assigned to evaluate study quality with 9–10 stars indicating “very good” quality, 7–8 stars “good” quality, 5–6 stars “satisfactory” quality, and 0–4 stars indicating “unsatisfactory” quality. Two authors (NA and GB) have conducted the quality appraisals and the average assessment scale of the two authors were used for the final decision.

### Data extraction and management

Data was extracted using Microsoft Excel 2016 spreadsheet and the Joanna Briggs Institute (JBI) data extraction form for observational studies. Two authors (GB and KD) reviewed the included studies and extracted data from eligible articles. The third reviewer (NE) handled disagreements between the two authors and consensus was reached through discussion.

### Heterogeneity

Heterogeneity was assessed using Cochran’s Q statistics and the I^2^ tests. A *p*-value < 0.10 of the Cochran’s Q statistic and the I^2^ test statistic of greater than 75% were declared to have significant statistical heterogeneity [29]. Significant heterogeneity was observed between studies; consequently, subgroup analysis was conducted based on the types of epilepsy reported, study settings, and by the region of the country. However, non of these were the source of heterogeneity.

### Publication bias

**Fig. 2:**
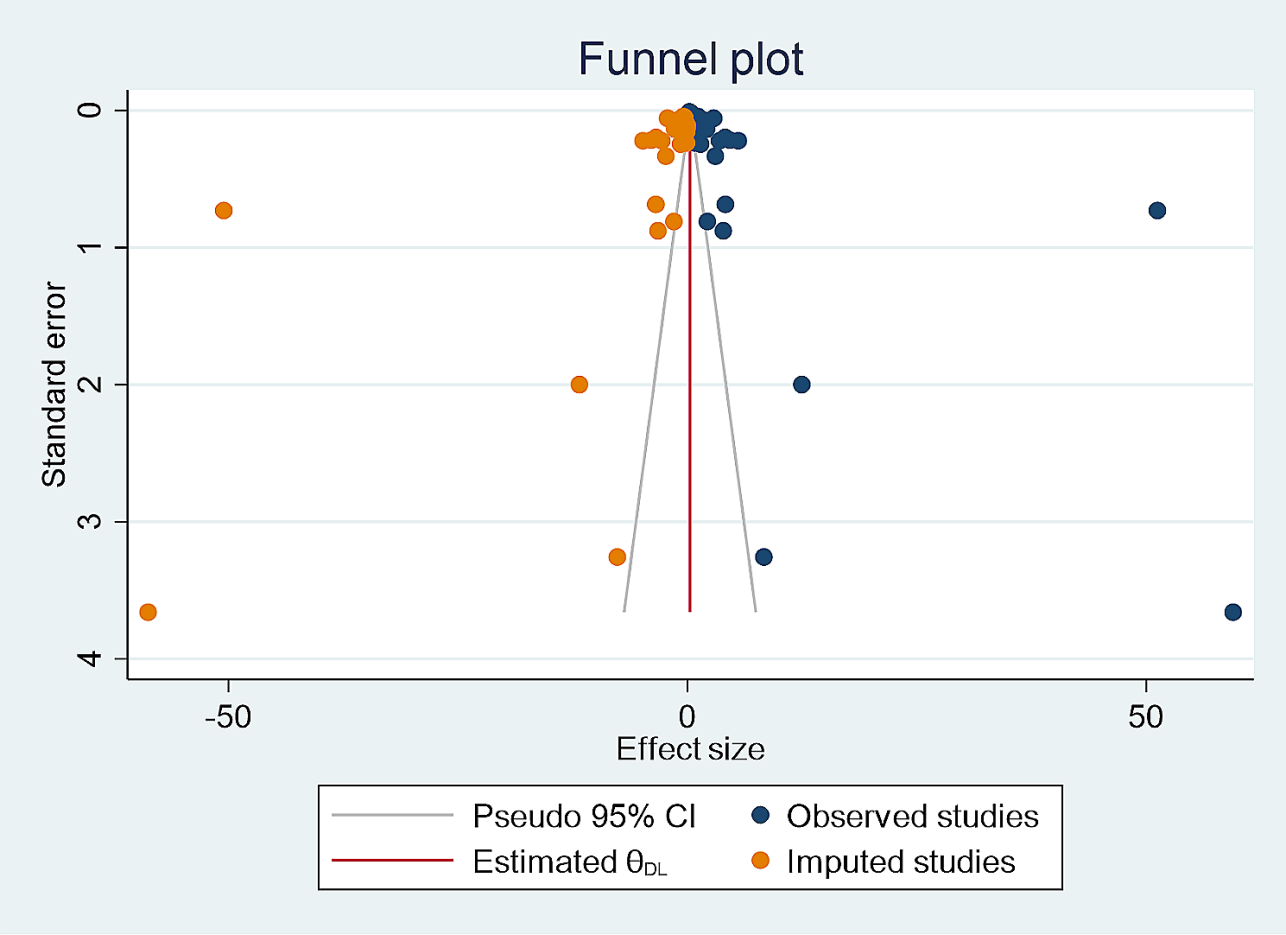
A Funnel plot that includes both the observed studies and the imputed studies

The possible risk of publication bias was examined by visual inspection of funnel plot and by the statistically by Begg’s correlation and Egger’s regression tests. The visual inspection of funnel plot showed significant publication bias with substantial asymmetry. Consequently, Begg’s correlation and Egger’s regression tests were performed. Egger’s and Begg’s statistical tests revealed significant publication bias with *p*-value of < 0.001 and *p* = 0.0034 respectively. Sensitivity analysis showed that individual studies have excessive influence on the overall estimate since the point estimate of omitted studies lies outside the confidence interval of the combined analysis. Due to the presence of a substantial publication bias, trim and fill analysis was performed which yields an unbiased estimate of effect size (Fig. 2).

## Result

### Study characteristics

A total of 42 studies with 56 findings (active, lifetime, or unclassified epilepsy) were included to estimate the pooled prevalence of epilepsy in children and adolescents aged less than 18 years. Of these included epilepsy findings, 26 were active epilepsy [17, 30–49] and 8 were lifetime epilepsy [35, 38, 39, 42, 44, 46, 50, 51]. However, the remaining 22 were not mentioned whether active or lifetime epilepsy and we reported it unclassified epilepsy [21, 34, 48, 52–69]. Thirty studies were population-based studies [17, 30, 32, 33, 35–44, 46–52, 56–58, 62, 66–70]; 5 hospital-based studies with 3 in neurological units [21, 53, 54, 59, 60]; and 7 studies were from high parasitic endemic areas [34, 45, 55, 61, 63–65] (Table 1).

**Table 1:** Characteristics of included studies the prevalence of epilepsy in African countries August 2023

Author (year)	Country	Design	Sample	Event	Quality	Age	Epilepsy type	Study setting
Abuga JA et al. (2001)	Kenya	survey	10,218	419	8	6–9 yrs	Unspecified epilepsy	Population based
Abuga JA et al. (2015)	Kenya	survey	11,223	193	8	6–9 yrs	Unspecified epilepsy	Population based
Abuga JA et al. (2022)	Kenya	cs	86,360	2466	7	5–14 yrs	Unspecified epilepsy	hospital based
Achermann et al. (2019)	S/Africa	ret	4701	2407	8	1–12 yrs	Unspecified epilepsy	neurological unit
Agbohoui O et al. (1999)	Senegale	cs	2803	58	7	3–10 yrs	active epilepsy	Population based
Angwafor SA et al. (2021)	Camerron	cs	16,489	157	8	6–19 yrs	active epilepsy	Population based
Balogou AAK et al. (2007)	Togo	cs	2772	29	7	< 15 yrs	active epilepsy	Population based
Bistervels IM et al. (2016)	Kenya	Cohort	16,438	238	8	≤ 13 yrs	Unspecified epilepsy	hospital based
Burton K et al. (2012)	Tanzania	cs	38,523	112	8	6–14 yrs	active epilepsy	community based
Carter JA et al. (2004)	Kenya	survey	487	19	7	6–9 yrs	active epilepsy	hospital based
Carter JA et al. (2004)	Kenya	survey	487	36	7	6–9 yrs	Unspecified epilepsy	hospital based
christianson AL et al. (2000)	S/Africa	cs	6692	49	7	2–9 yrs	life time epilepsy	community based
christianson AL et al. (2000)	S/Africa	cs	6692	45	7	2–9 yrs	active epilepsy	community based
Colebunders R et al. (2018	S/Sudan	survey	10,696	591	8	< 20 yrs	Unspecified epilepsy	community based
Dent W et al. (2005)	Tanzania	survey	2592	29	8	≤ 19 yrs	active epilepsy	population based
Dossou GA et al. (2003)	Benin	cs	1400	11	7	< 20 yrs	Unspecified epilepsy	community based
Duggan MB et al. (2010)	Uganda	survey	193,126	395	6	≤ 15 yrs	Unspecified epilepsy	community based
Edward T et al. (2007)	Kenya	survey	67,008	217	8	6–17 yrs	active epilepsy	Population based
Eseigbe E et al. (2014	Nigeria	survey	3613	23	6	≤ 18 yrs	Unspecified epilepsy	Population based
Eyong KI et al. (2017)	Nigeria	cs	180	107	6	≤ 18 yrs	Unspecified epilepsy	neurological unit
Ezeala-Adikaibe et al. (2016)	Nigeria	cs	1211	4	7	15–19 yrs	active epilepsy	community based
Ezeala-Adikaibe et al. (2016)	Nigeria	cs	1211	39	7	15–19 yrs	life time epilepsy	community based
Farghaly WM et al. (2018)	Egypt	cs	36,195	350	6	≤ 18 yrs	life time epilepsy	Population based
Frank-Briggs A et al. (2011)	Nigeria	ret	35,473	584	6	3–15 yrs	Unspecified epilepsy	neurological unit
Houinato D et al. (2013)	Benin	cs	6874	41	7	≤ 19 yrs	life time epilepsy	community based
Houinato D et al. (2013)	Benin	cs	6874	10	7	< 20 yrs	active epilepsy	community based
Hunter et al. (2012	Tanzania	cc	16,762	42	7	15–19 yrs	active epilepsy	community based
Ibinda F eta al (2014)	Kenya	cs	128,339	419	8	≤ 18 yrs	active epilepsy	community based
Kakooza A et al. (2017)	Uganda	survey	64,172	152	8	≤ 18 yrs	active epilepsy	Population based
Kariuki SM et al. (2021)	Kenya	cs	2660	81	7	≤ 18 yrs	life time epilepsy	Population based
Kariuki SM et al. (2021)	Kenya	cs	2660	56	7	≤ 18 yrs	active epilepsy	Population based
Kariuki SM et al. (2015)	Kenya	cs	128,344	1004	7	≤ 18 yrs	active epilepsy	Population based
Kariuki SM et al. (2015)	S/Africa	cs	36,916	162	7	≤ 18 yrs	active epilepsy	Population based
Kariuki SM et al. (2015)	Uganda	cs	37,138	460	7	≤ 18 yrs	active epilepsy	Population based
Kariuki SM et al. (2015)	Tnzania	cs	48,066	504	7	≤ 18 yrs	active epilepsy	Population based
Kariuki SM et al. (2015)	Gana	cs	57,564	660	7	≤ 18 yrs	active epilepsy	Population based
Kind CJ et al. (2017)	Kenya	cs	11,223	234	8	6–9 yrs	life time epilepsy	Population based
Kind CJ et al. (2017)	Kenya	cs	11,223	129	8	6–9 yrs	active epilepsy	Population based
Lenaerts E et al. (2018)	Congo	cs	846	35	8	≤ 18 yrs	active epilepsy	community based
Levick B et al. (2017)	Congo	survey	6934	242	9	≤ 18 yrs	Unspecified epilepsy	Population based
Mahmoud NAH et al. (2009)	Egypt	cs	8750	63	7	6–12 yrs	life time epilepsy	Population based
Mohamed IN et al. (2017)	Sudan	cs	74,949	303	6	6–14 yrs	Unspecified epilepsy	Population based
Mung’ala-Odera V (2008)	Kenya	survey	10,218	419	8	6–9 yrs	life time epilepsy	Population based
Mung’ala-Odera V (2008)	Kenya	survey	10,218	112	8	6–9 yrs	active epilepsy	Population based
Ngoungou EB et al. (2006)	Mali	cs	323	7	9	≤ 15 yrs	Unspecified epilepsy	hospital based
Ngugi et al. (2003)	Nigeria	cs	66,998	218	8	6–18 yrs	active epilepsy	Population based
Ngugi et al. (2003)	Nigeria	vs.	129,069	379	8	≤ 18 yrs	active epilepsy	Population based
Nwani PO et al. (2015)	Nigeria	cs	3148	16	8	≤ 19 yrs	Unspecified epilepsy	Population based
Nwani PO et al. (2015)	Nigeria	cs	3148	20	8	≤ 19 yrs	active epilepsy	Population based
Prischich F (2008)	Camerron	cs	72	6	8	≤ 19 yrs	Unspecified epilepsy	Population based
Raimon et al. (2021)	S/Sudan	Survey	9411	436	7	< 20 yrs	Unspecified epilepsy	Population based
Rwiza HT et al. (1992)	Tanzania	cs	11,023	73	8	≤ 19 yrs	active epilepsy	community based
Sebera F et al. (2005)	Rwanda	cs	273	34	7	≤ 19 yrs	Unspecified epilepsy	Population based
Simms V et al. (2008)	Rwanda	cs	3212	25	7	≤ 15 yrs	Unspecified epilepsy	Population based
Winkler AS et al. (2009)	Tanzania	survey	4316	32	8	≤ 19 yrs	Unspecified epilepsy	Population based
Yemadje LP et al. (2012)	Benin	survey	2371	34	8	15–19 yrs	Unspecified epilepsy	Population based

The highest prevalence of childhood epilepsy was observed in neurological units of hospital-based studies in South Africa and Nigeria. In these studies more than half children who visited the neurological units had epilepsy [53, 59]. In addition, a higher prevalence of epilepsy was reported in parasitic endemic areas of Cameroon, the Republic of Sudan, South Sudan, and the Republic of Congo [45, 55, 61, 64, 65]. Additionally, exposure to multiple parasites is associated with a higher rate of active convulsive epilepsy [71].

### Prevalence of epilepsy

**Fig. 3:**
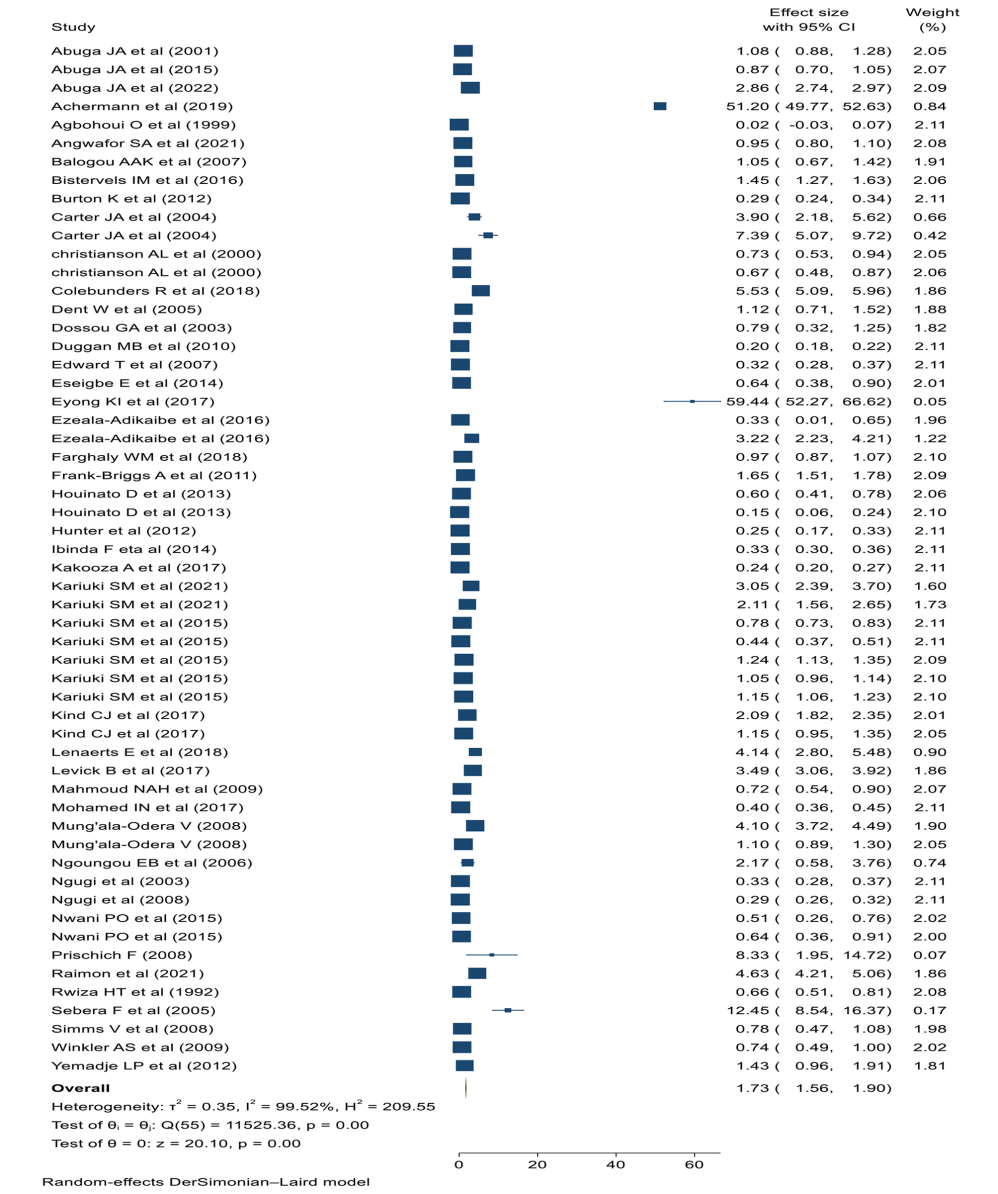
Forest plot of the pooled childhood epilepsy in Africa

**Fig. 4:**
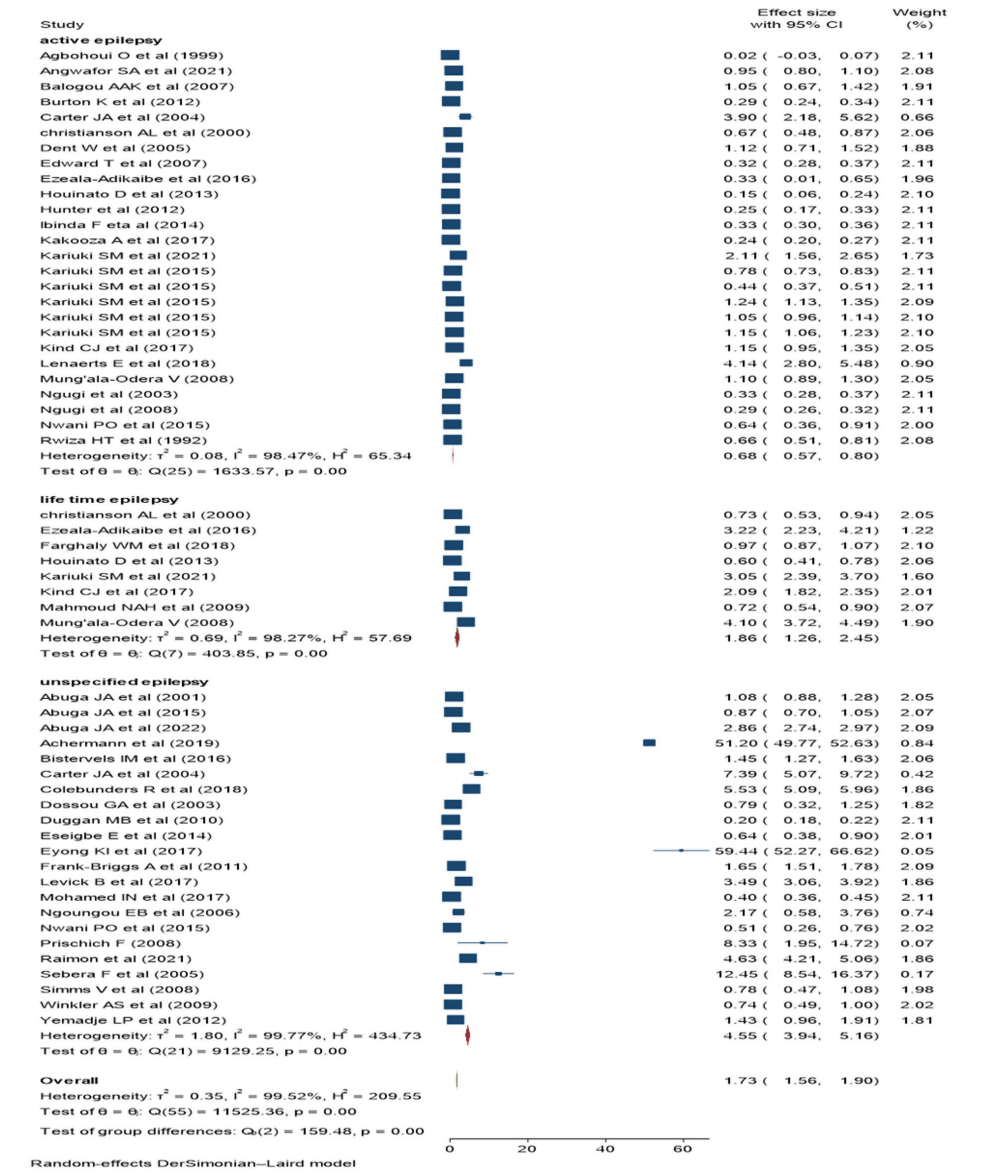
Sub group analysis of childhood epilepsy by the types of epilepsy

The pooled prevalence of cumulative epilepsy was 17.3 per 1000 children (95% CI: 15.60–19.00, I^2^: 99.52%, *p* < 0.001) (Fig. 3). Significant heterogeneity was observed between studies, consequently, subgroup analysis was performed between types of epilepsy (active, lifetime or unclassified), study settings (institutional, population based, or high parasite endemic areas), and the region or country location (northern, central, eastern, or southern Africa). The pooled prevalence of lifetime and active epilepsy were 18.6 (95% CI: 12.6–24.5, I^2^: 98.27%, *p* < 0.001) and 6.8 (95%CI: 5.7-8 per; I^2^: 98.47%, *p* < 0.001) per 1000 children respectively. On the other hand, the pooled prevalence of unclassified epilepsy was 45.5 (95% CI: 39.4–61.6, I^2^: 99.77%, *p* < 0.001) per 1000 children (Fig. 4).

**Fig. 5:**
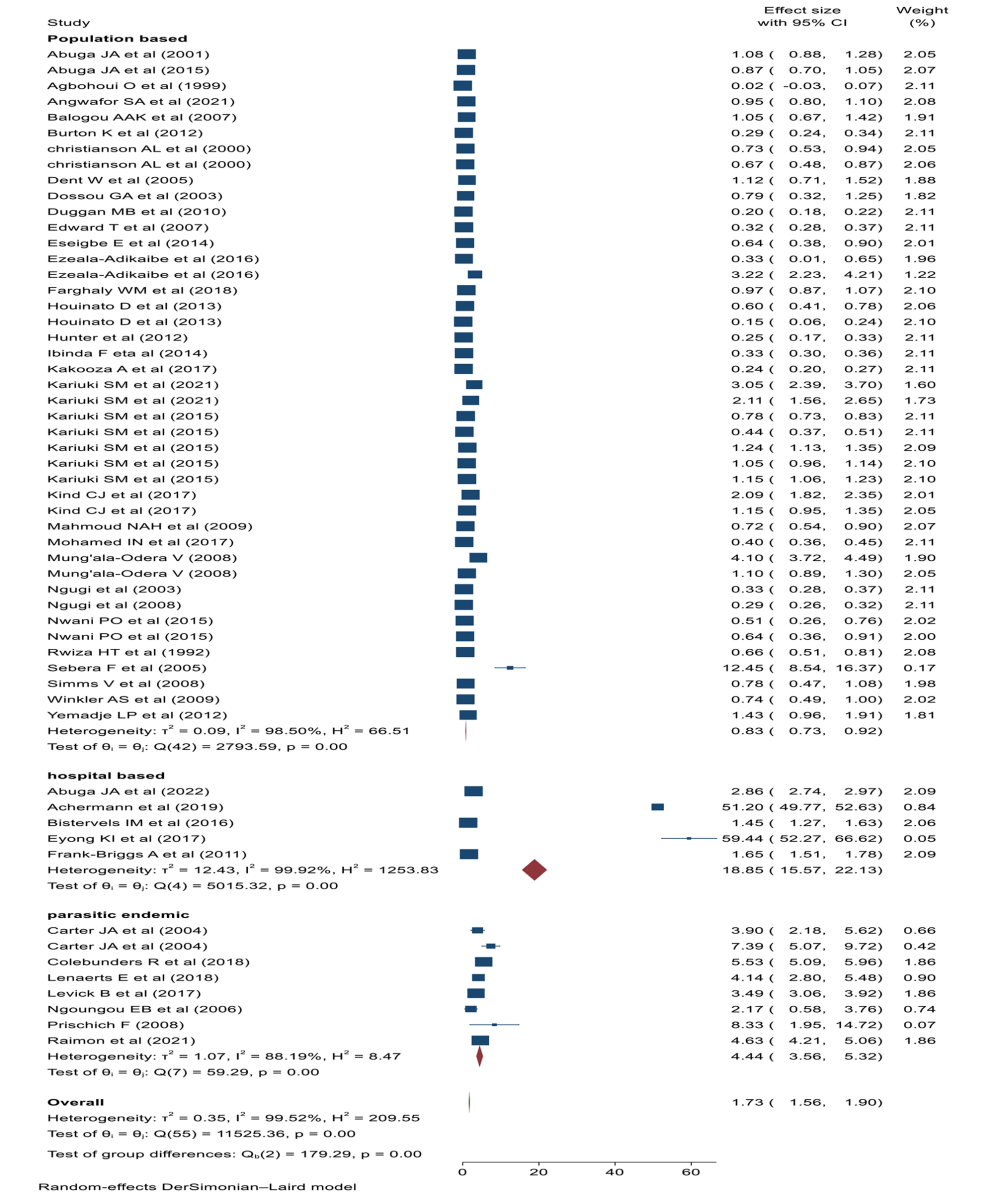
Sub-group analysis of childhood epilepsy by the study setting

**Fig. 6:**
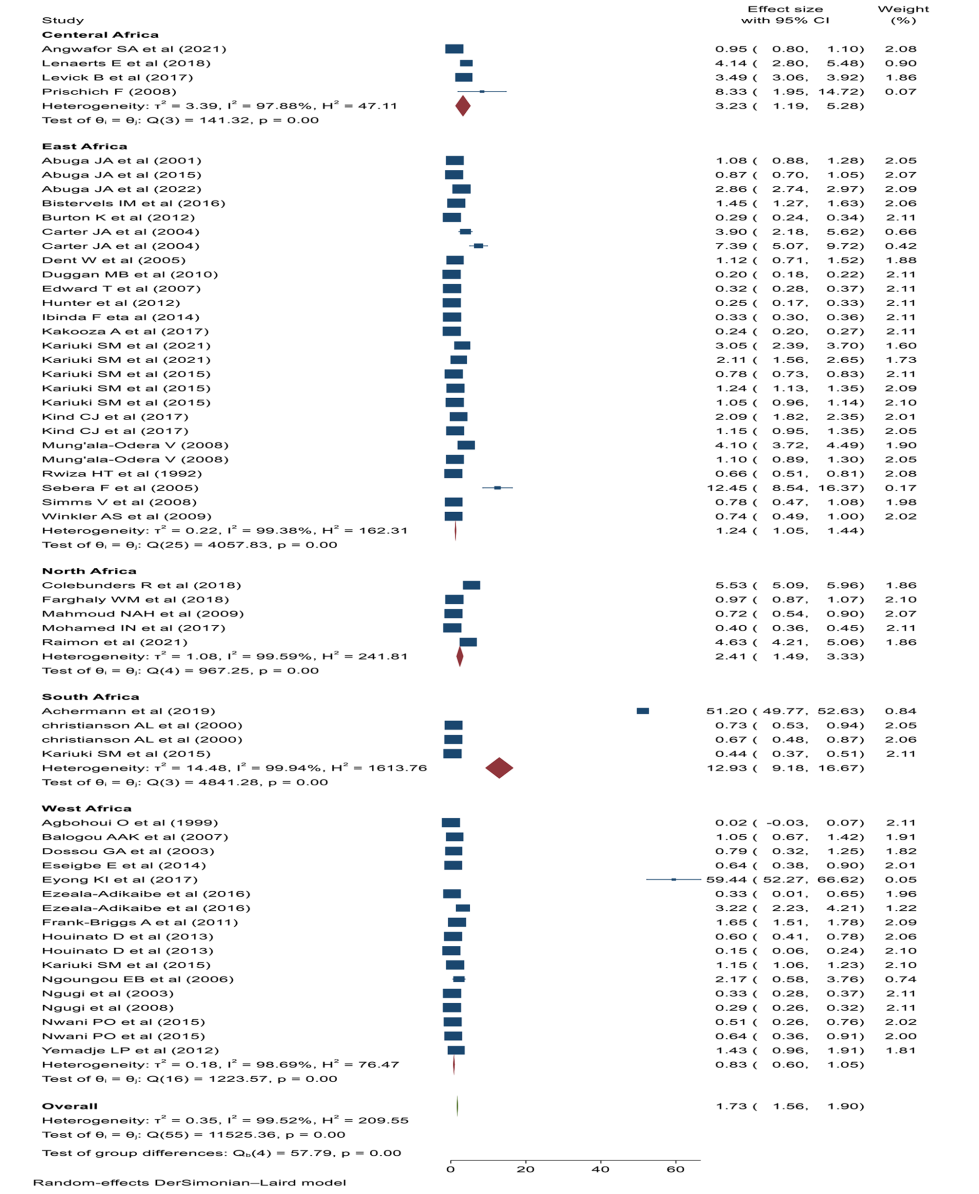
Sub group analysis by the region

The pooled prevalence of epilepsy in the health institutions was 189 (95% CI: 155.7, 221.3) per 1000 children. Whereas, the pooled prevalence of epilepsy in high parasite endemic areas was 44 (95%CI: 35.6–53.2) per 1000 children. However, the pooled prevalence of epilepsy in the general population was 8 (95% CI: 7.3 to 9.2) per 1000 children (Fig. 5). Subgroup analysis was performed by region or location of the country. The highest prevalence of epilepsy was reported in Southern African countries (129.3/1000) followed by Central African countries (32.3/1000) and Northern African countries (24.1/1000 (Fig. 6).

### Incidence of epilepsy

**Fig. 7:**
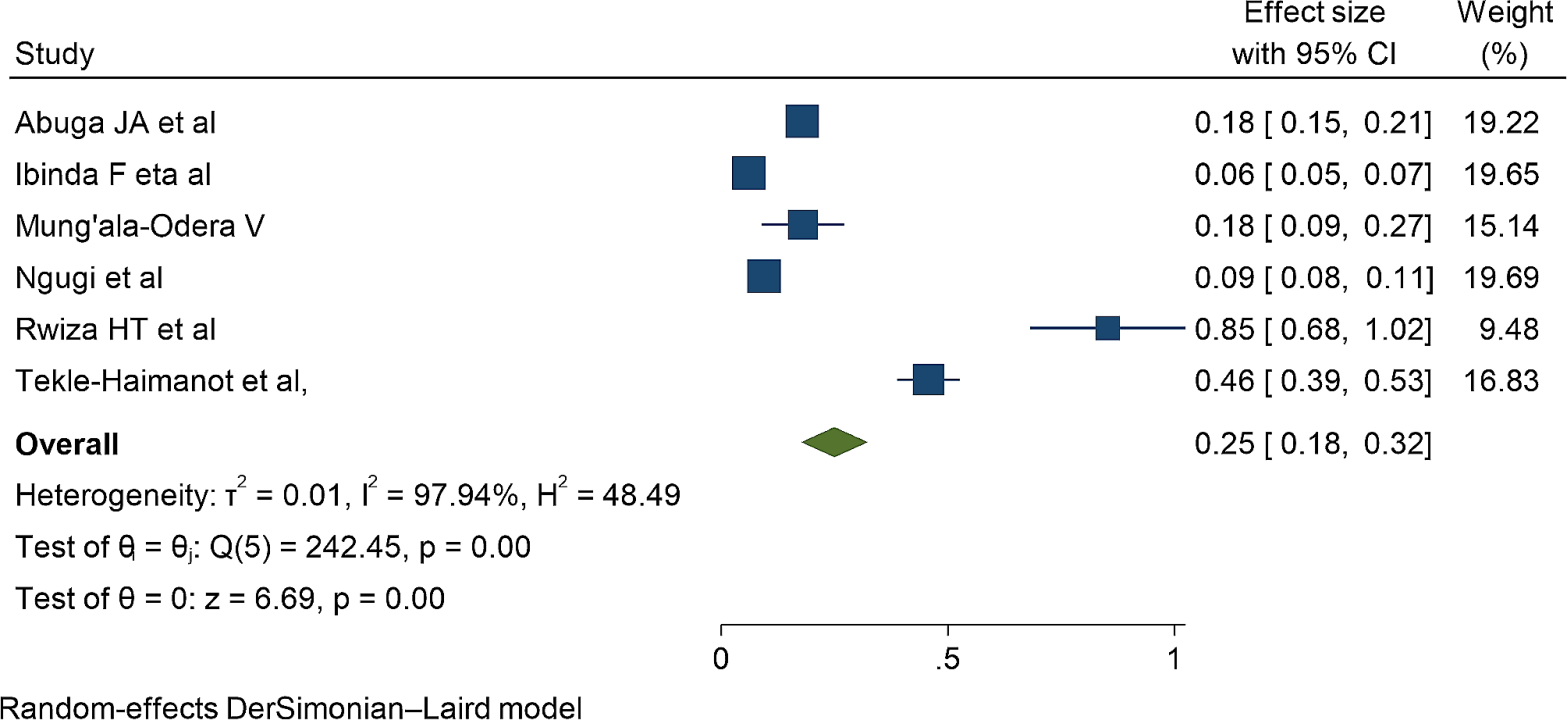
Forest plot for the pooled incidence of childhood epilepsy in Africa

Six studies were included to estimate the pooled incidence of epilepsy in children and adolescents in Africa. The incidence of epilepsy varies significantly in the African countries. The highest incidence was reported in Tanzania (850 per 100,000) [72] and the lowest reported in Kenya (60/100,000) [41] and Nigeria (94/100,000) [73]. The pooled incidence of epilepsy in children and adolescents was 250 per 100, 000 children (95%CI:180–320, I^2^: 97.94%, *p* < 0.001)(Fig. 7).

## Discussion

In this review and meta-analysis, the authors explored and integrate evidence available on childhood epilepsy in Africa. Through gathering and summarizing all available evidence on childhood epilepsy the authors have provided a more representative and reliable data regarding childhood epilepsy in the continent. The knowledge derived from this review and meta-analysis can be used as baseline data by policymakers, national and international organizations, researchers, and other stakeholders to design and implement strategies to control childhood epilepsy in Africa.

The prevalence of epilepsy varies significantly between and within countries in Africa. The prevalence rate ranges from 0.2/1000 in Senegal [30] to 510/1000 in South Africa [53] and 594/1000 in Nigeria [59]. This variation could be attributed to the heterogeneity of methodology or differences in the definition of epilepsy between studies. The variation could also be due to the nature of reported epilepsies; lifetime, active, or cumulative epilepsies. In addition, the variation could be related to the settings in which the samples selected. In some studies, samples were selected from the general population which could better represent the population. However, in other studies, samples were selected from hospitals particularly in neurological units which increases the prevalence. Additionally, the inconsistencies might be related to the use of different epilepsy screening tools.

This study reveals that epilepsy remains a significant public health concern in Africa. One in fifty children in Africa are suffering from this preventable and treatable medical condition. This finding is supported by the global studies [11, 74] and studies in developing countries [10, 13, 18]. The reason could be children are vulnerable to perinatal and neonatal complications and early childhood infections which are risk factors of epilepsy [10, 43]. Moreover, the high endemicity of neuro-parasites in some African countries might contribute to the high burden of epilepsy in children and adolescents in Africa [71].

The prevalence of cumulative epilepsy was 17.3 per 1000 children (95% CI: 15.60–19.00). This finding is higher than the global prevalence of epilepsy (9·39/1000, 95% CI: 8·55–10·23) [12]. The reason could be explained in terms of population differences where the current study involved children and adolescents, which increases the prevalence of epilepsy due to the neonatal and perinatal complications as well as early childhood infections. The prevalence of active epilepsy was 6.8 (95%CI: 5.7–8.00) per 1000 children. The finding is congruent with the systematic review and meta-analysis of international studies (6.38, 95% CI: 5.57–7.30) per 1000 persons [6]. The high prevalence of active epilepsy in children and adolescents could be related to the poor treatment adherence or frequent exposure to epilepsy risk factors.

The prevalence of lifetime epilepsy was 18.6 (95% CI: 12.6–24.5 ) per 1000 children. The finding was higher than the systematic review and meta-analysis of international studies (7.60, 95% CI 6.17–9.38) per 1000 children [6] and with similar estimate of the global burden of epilepsy [7]. The reason could be partly explained in terms of the higher reported risk factors of epilepsy in Africa such as perinatal and neonatal infection, traumatic brain injury, and other central nervous system infections. In addition, the large treatment gap and poor treatment outcome contribute to the high burden of epilepsy in the African countries [17, 58].

The incidence rate of epilepsy is highly varied across studies [41, 72, 73]. The observed heterogeneity could be attributed to the incidence rate estimation where some studies estimated the incidence rate per year and others estimated in every two or three years. The pooled incidence of epilepsy was 250 (95%CI:180–320, I^2^: 97.94%, *p* < 0.001) per 100,000 children. The incidence was higher than global incidence of epilepsy (89.06, 95%CI; 31.68– 98.01) [21], the meta-analysis of international studies (61.44, 95% CI 50.75–74.38) per 100,000 person-years [6], and a study in Norway (70, 95%CI: 64–75) [16]. The reason could be explained by the different in the source populations at risk where the current study involves children and adolescents whereas the later study was on the general population.

The prevalence of epilepsy was 24 folds higher in institutional based studies compared to population based studies. The reason could be children in neurological units are complaints of neurological problems which increases the prevalence of epilepsy due to the higher epilepsy case load in these institutions. The higher prevalence of epilepsy in the health institutions reveals that emphasis should be given to mass epilepsy screening at the community level.

The pooled estimate of childhood epilepsy was higher in parasite endemic areas. This finding is supported by other studies in the continent [71, 75]. The reason could be neuro-parasites causes infection to the brain which then alter the electrical activity of the brain and causes epilepsy [71]. Therefore, prevention and early treatment of parasitic infections such as malaria, neurocysticercosis, and onchocerca has an advantage to the control of childhood epilepsy.

**Fig. 8:**
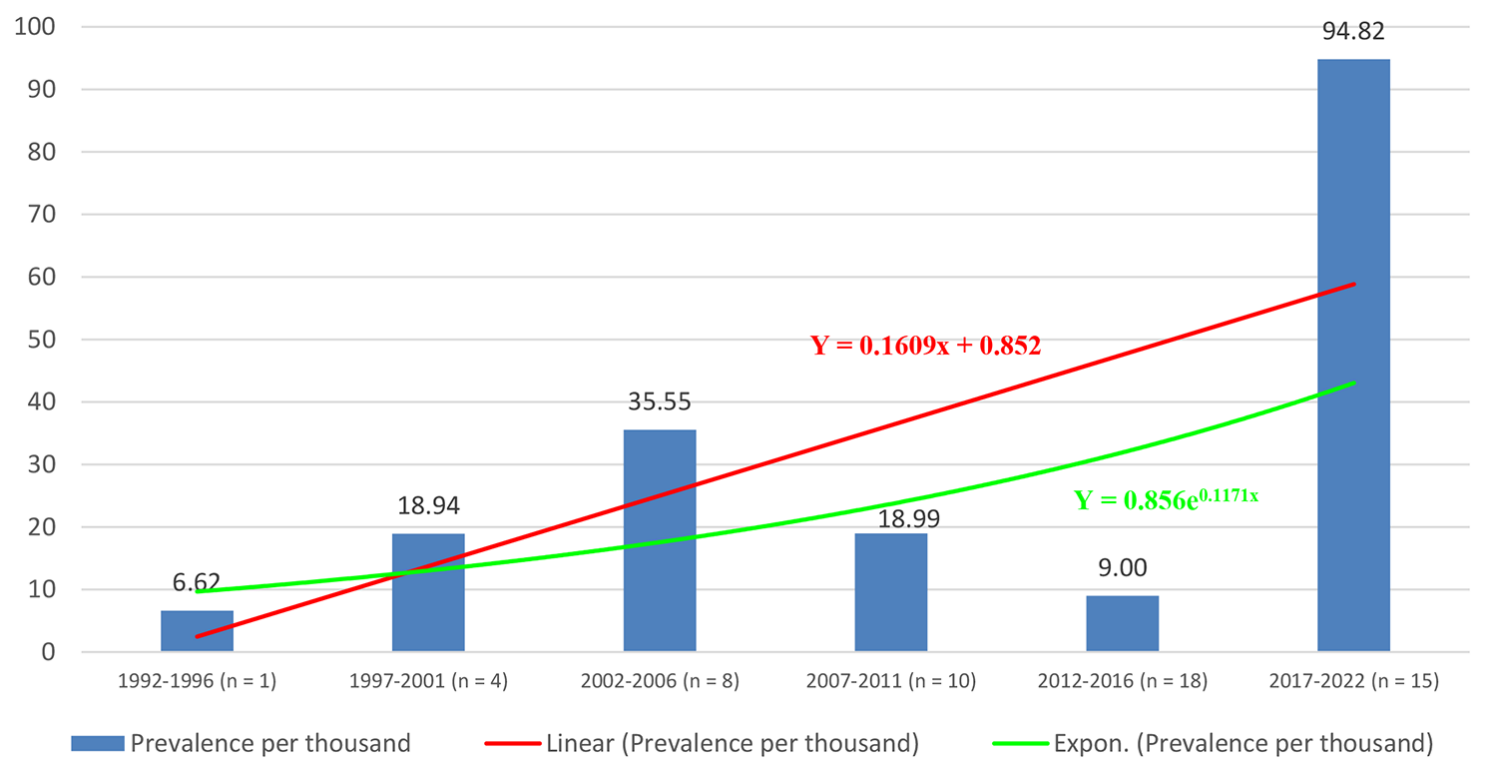
Trends of childhood epilepsy prevalence in Africa from 1992 to 2022 (n = number of study in the specified years)

This study reveals that the prevalence of epilepsy has been increasing over the last 30 years in children and adolescents. This could be due to the fact that the number of studies on epilepsy increases over time which could better investigate more epilepsy cases in the community. In addition, the increased prevalence may actually reflect better awareness and education around epilepsy due to increased efforts in the past decade. Thus, there may be both better recognition by providers as well as better health-seeking behaviors. The highest prevalence of epilepsy was reported between 2017 and 2022. The average rate of epilepsy in these years was 19.82 per 1000 children. The reason could be explained in terms of increased population crisis and its associated negative mental health consequences which might contribute to an increased burden of epilepsy. This suggests that more population-based epilepsy screening as well as escalating the prevention and treatment of epilepsy should be strengthened (Fig. 8).

## Conclusion

Epilepsy has still contributed to a significant disease burden in children and adolescents in African. However, little or no attention has been paid to the prevention and control of the diseases. Mass epilepsy screening, escalating preventive and treatment measures, as well as regular treatment follow up and monitoring are recommended towards the control of childhood epilepsy.

## Data Availability

Data will be available from the corresponding author upon reasonable request.

## Abbreviations

HICs: High-Income Countries
LMICs: Low and Middle-Income Countries
NOS: Newcastle-Ottawa Quality Assessment Scale
PRISMA: Preferred Reporting Item for Systematic Review and Meta analysis
SSC: Sub Saharan Countries
